# Serum bone-turnover biomarkers are associated with the occurrence of peripheral and axial arthritis in psoriatic disease: a prospective cross-sectional comparative study

**DOI:** 10.1186/s13075-017-1417-7

**Published:** 2017-09-21

**Authors:** Deepak R. Jadon, Raj Sengupta, Alison Nightingale, Hui Lu, Juliet Dunphy, Amelia Green, James T. Elder, Rajan P. Nair, Eleanor Korendowych, Mark A. Lindsay, Neil J. McHugh

**Affiliations:** 1 Department of Rheumatology, Royal National Hospital for Rheumatic Diseases, Bath, UK; 2 Department of Rheumatology, Cambridge University Hospitals NHSFT, Cambridge, UK; 3 Department of Pharmacy & Pharmacology, University of Bath, Bath, UK; 4 Department of Dermatology, Ann Arbor Veterans Affairs Hospital, Ann Arbor, Michigan USA

**Keywords:** Biomarkers, Psoriatic arthritis, Ankylosing spondylitis, Psoriasis, Spondylitis, Dkk-1, MMP-3, M-CSF, Osteoprotegerin

## Abstract

**Background:**

A recent systematic review identified four candidate serum-soluble bone-turnover biomarkers (dickkopf-1, Dkk-1; macrophage-colony stimulating factor, M-CSF; matrix metalloproteinase-3, MMP-3; osteoprotegerin, OPG) showing possible association with psoriatic arthritis (PsA). We aimed to: (i) confirm and determine if these four biomarkers are associated with PsA; (ii) differentiate psoriasis cases with and without arthritis; and (iii) differentiate PsA cases with and without axial arthritis.

**Methods:**

A prospective cross-sectional comparative two-centre study recruited 200 patients with psoriasis without arthritis (PsC), 127 with PsA without axial arthritis (pPsA), 117 with PsA with axial arthritis (psoriatic spondyloarthritis, PsSpA), 157 with ankylosing spondylitis (AS) without psoriasis, and 50 matched healthy controls (HC). Serum biomarker concentrations were measured using ELISA. Multivariable regression and receiver operating characteristic analyses were performed.

**Results:**

MMP-3 concentrations were significantly higher and M-CSF significantly lower in each arthritis disease group compared with HC (*p* ≤ 0.02). MMP-3 concentrations were significantly higher (adjusted odds ratio, OR_adj_ 1.02 per ng/ml increase in concentration; *p* = 0.0004) and M-CSF significantly lower (OR_adj_ 0.44 per ng/ml increase; *p* = 0.01) in PsA (pPsA and PsSpA combined) compared with PsC. Dkk-1 concentrations were significantly higher (OR_adj_ 1.22 per ng/mL increase; *p* = 0.01), and OPG concentrations significantly lower (OR_adj_ 0.20 per ng/mL increase; *p* = 0.02) in patients with axial arthritis (PsSpA and AS combined) than in those without (pPsA). Furthermore, Dkk-1 concentrations were significantly higher along a spectrum of increasing axial arthritis; Dkk-1 concentrations were higher in AS compared with PsSpA (OR_adj_ 1.18 per ng/mL increase; *p* = 0.02). Receiver operating characteristic analysis showed MMP-3 to be the best single biomarker for differentiating PsA from PsC (AUC 0.70 for a cut-off of 14.51 ng/mL; sensitivity 0.76, specificity 0.60).

**Conclusions:**

MMP-3 and M-CSF are biomarkers for the presence of arthritis in psoriatic disease, and could therefore be used to screen for PsA in psoriasis cohorts. Dkk-1 and OPG are biomarkers of axial arthritis; they could therefore be used to screen for the presence of axial disease in PsA cases, and help differentiate PsSpA from AS. High concentrations of Dkk-1 in AS and PsSpA compared with HC, support previous reports that Dkk-1 is dysfunctional in the spondyloarthritides.

## Background

Psoriatic arthritis (PsA) and ankylosing spondylitis (AS) are chronic inflammatory conditions of the musculoskeletal system belonging to the family of spondyloarthritis (SpA). They are characterised by bone resorption in the form of erosion and osteolysis, and new bone formation (osteoproliferation) in the form of syndesmophytes, periostitis and sacroiliac joint (SIJ) ankylosis. The bone forming and resorbing phenotypes of PsA and AS make them good candidates to investigate the role of serum-soluble bone-turnover biomarkers.

A systematic literature review of serum-soluble bone and cartilage-turnover markers in PsA and psoriatic spondyloarthritis (PsSpA) [1], identified four candidate bone-turnover markers in PsA: dickkopf 1 (Dkk-1), which inhibits Wnt-mediated bone formation; osteoprotegerin (OPG), which inhibits RANK-mediated bone resorption; macrophage colony stimulating factor (M-CSF),which promotes bone resorption; and matrix metalloproteinase 3 (MMP-3), which degrades the extra-cellular matrix of bone and cartilage, leading to bone erosion and joint space narrowing.

We have previously reported that the presence of psoriasis and *HLA-B27* are important factors that define the pattern of axial disease in SpA [2]. We now hypothesise that serum-soluble bone-turnover biomarkers are associated with disease phenotype and disease severity in patients with SpA. Using the same cohort and comparator samples from patients with psoriasis only (cases) and matched healthy controls, the aims of this study were to confirm and determine if these four biomarkers (Dkk-1, M-CSF, MMP-3, and OPG): (i) are associated with PsA; (ii) differentiate patients with psoriasis with and without arthritis; and (iii) differentiate patients with PsA with and without axial arthritis.

## Methods

### Study participants

A prospective cross-sectional comparative two-centre study (Axial Disease in Psoriatic Arthritis study; ADIPSA) was performed, recruiting unselected consecutive patients with PsA and AS from a secondary-care teaching hospital attended by approximately 600 patients with PsA and 700 with AS between August 2012 and October 2013. The study inclusion criteria were: patients aged ≥ 18 years with either PsA fulfilling the Classification of Psoriatic Arthritis (CASPAR) criteria [3, 4] or AS fulfilling the 1987 modified New York diagnostic criteria (mNYc) for AS [5]. The enrolled patients with PsA and AS were reclassified as: peripheral-only PsA (pPsA) without radiographic axial disease (RAD), PsA with RAD (psoriatic spondyloarthritis; PsSpA), and AS without psoriasis (AS). In keeping with previous publications [6, 7], inflammatory RAD was defined in patients with PsSpA as the presence of: New York criteria sacroiliitis (unilateral grade ≥ 3, or bilateral grade ≥ 2 sacroiliitis), and/or ≥ 1 marginal/paramarginal syndesmophyte(s) of the cervical or lumbar spine. The most recent (≤10 years) axial radiographs of all participants with SpA were scored using two validated indices: the Psoriatic Arthritis Spondylitis Radiology Index (PASRI) [8] and the modified Stoke Ankylosing Spondylitis Spinal Score (mSASSS) [9].

At the time of blood sample collection all study participants were assessed by a rheumatologist (DRJ) who collected data on patient-reported outcome measures (PROMs), clinical examination indices, and axial radiographic indices. Further details on the ADIPSA cohort have been published previously [2].

Serum from 200 patients with purely cutaneous psoriasis (PsC) without clinical evidence of joint problems for ≥ 10 years since the diagnosis of psoriasis, assessed by a dermatologist, were provided from the Department of Dermatology (University of Michigan), for whom associated clinical details including sex, age, disease duration, biologic and synthetic disease-modifying anti-rheumatic drug (DMARD) use, and Psoriasis Area Severity Index (PASI) were available. Serum from 50 healthy controls (HC) was obtained from the UK Health and Social Care Information Centre (HSIC), the controls having been deemed healthy using a screening questionnaire. HCs were age, sex and ethnicity matched to the enrolled patients with PsA.

### Serum biomarker testing

Patients provided non-fasted blood samples on the morning of clinical assessment and serum was stored at − 80 °C, with minimal freeze-thaw cycles [10]. Commercially available enzyme-linked immunosorbent assay (ELISA) kits were used to measure biomarker concentrations in each subject group in duplicate. Quantikine ELISA kits (R&D systems, UK) were used to measure total Dkk-1 (functionally active and inactive Dkk-1), total MMP-3 (pro-MMP-3 and active-MMP-3), and total M-CSF. Another ELISA kit (Biorbyte, UK) was used to measure total OPG. A percentage coefficient of variation (CV%) ≤ 15% between duplicate samples was considered acceptable, and samples were re-tested until CV% ≤ 15% was attained.

### Statistical analysis

A statistical analysis plan was decided a priori, guided by the systematic review of biomarkers [1] and mechanistic hypotheses. Adjustment for multiple testing was therefore not required. Data were analysed using STATA12.1 (2011 TX, USA). Since serum biomarker concentrations were not normally distributed (skewed to the right, at group and cohort level), a logarithmic transformation was applied prior to all analyses. Reverse, stepwise, logistic regression, adjusted for covariables at blood sampling (sex, age, disease duration from diagnosis, anti-TNF use, body mass index (BMI), AS Disease Activity Score (ASDAS), and PASI) was used to compare biomarker concentrations between subject groups. The alpha level for statistical significance was 0.05.

## Results

The study enrolled 651 subjects: 200 with PsA, 201 with AS, 200 with PsC and 50 HC. The PsA and AS cases were reclassified as: 127 pPsA, 117 PsSpA, and 157 AS (43 cases of psoriasis, but meeting both modified New York criteria for AS and CASPAR criteria for PsA, were reclassified as PsSpA for this research study). The clinical characteristics of the groups are detailed in Table 1. Axial radiographs were mostly recent (median interval between radiographs being performed and study enrolment 2.4 years, IQR 1.4, 4.1). Excellent inter-rater (intraclass correlation coefficient (ICC) ≥ 0.85) and intra-rater (ICC ≥ 0.88) reliability was achieved by raters for the PASRI, mSASSS and regional subdomains. For brevity, further clinical, radiographic and treatment characteristics of this cohort are described in the clinical-radiographic paper on the ADIPSA cohort [2].

**Table 1:** Demographic and clinical characteristics of the healthy control and disease groups

At blood sampling	HC (*n* = 50)	PsC (*n* = 200)	pPsA (*n* = 127)	PsSpA (*n* = 117)	AS (*n* = 157)
	Median (IQR)	Median (IQR)	Median (IQR)	Median (IQR)	Median (IQR)
Age (years)	59.93 (51.44, 67.56)	54 (42, 65)	58.45 (50.31, 66.63)	59.53 (59.56, 66.54)	54.70 (44.52, 63.41)
Disease duration (years)	n/a	24 (16, 36.5)	15 (7, 26)	18 (9, 27)	22 (10, 31)
BMI (kg/m^2^)	n/a	n/a	29.04 (26.30, 32.84)	28.56 (26.06, 31.98)	27.07 (24.58, 30.13)
CRP (mg/dL)	n/a	n/a	2 (0, 5)	3 (2, 7)	4 (1, 10)
ASDAS (units)	n/a	n/a	2.1 (1.3, 2.7)	2.2 (1.5, 2.9)	2.1 (1.6, 2.9)
PASI (units)	n/a	4.8 (2.4, 8.8)	0.8 (0.0, 2.6)	0.8 (0.0, 2.8)	n/a
	*n* (%)	*n* (%)	*n* (%)	*n* (%)	*n* (%)
Male sex	26 (52.00)	102 (51.00)	66 (51.97)	74 (63.25)	118 (75.16)
Anti-TNF use	n/a	26 (13.00)	47 (37.01)	53 (45.30)	59 (37.58)
*HLA-B27* positive	n/a	n/a	9 (7.09)	47 (40.17)	140 (89.17)

*HC* healthy control, *PsC* psoriasis without arthritis, *pPsA* peripheral PsA, *PsSpA* psoriatic spondyloarthropathy, *AS* ankylosing spondylitis, *IQR* interquartile range, *n* number/proportion, *n/a* not available/applicable, *BMI* body mass index, *CRP* C-reactive protein, *ASDAS* AS Disease Activity Score, *PASI* Psoriasis Area Severity Index, *HLA* human leucocyte antigen, *Anti-TNF* anti-tumour necrosis factor drug, *sDMARD* synthetic disease modifying anti-rheumatic drug

### Assay reliability

Each of the four biomarkers achieved an acceptable inter-assay CV% (≤15%). However, despite several runs, 48 of 651 duplicate OPG samples did not attain an acceptable intra-assay CV% ≤ 10, and were excluded from subsequent analyses (14/157 AS, 1/118 PsSpA, 19/127 pPsA, 6/200 PsC, 9/50 HC).

### Biomarker concentrations in the cohort and analysis of covariables

Serum biomarker concentrations are detailed in Table 2. Linear and logistic regression were used to determine if biomarker concentrations were affected by the following covariables at blood sampling: sex, age, disease duration, anti-TNF use, BMI, *HLA-B27* positivity, high-sensitivity C-reactive protein (hsCRP), and ASDAS. The covariables included in the final regression models are given in Table 2 *.* MMP-3 (regression coefficient, ß 0.01; *p* = 0.04) and OPG (ß 0.01; *p* < 0.0001) concentrations significantly increased with age.

**Table 2:** Serum bone biomarker concentrations in disease groups compared with the reference healthy control group

Group	Serum concentration ng/mL Median (IQR)	Adjusted odds ratio* (per ng/mL increase in concentration)	95% CI	*p* value
**Dkk-1** (^Homogeneity *p* value = 0.08)				
HC	3.52 (2.72, 4.46)			
PsC	2.50 (1.87, 3.42)	0.72	0.61, 0.86	2 × 10^−4^
pPsA	3.03 (1.93, 3.69)	0.88	0.74, 1.04	0.14
PsSpA	3.34 (2.43, 4.44)	1.00	0.87, 1.15	0.99
AS	3.51 (2.92, 4.58)	1.10	0.97, 1.25	0.13
**M-CSF** (^Homogeneity *p* value = 0.26)				
HC	0.81 (0.41, 0.99)			
PsC	0.62 (0.25, 0.88)	0.59	0.32, 1.08	0.09
pPsA	0.29 (0.16, 0.68)	0.14	0.06, 0.32	<1 × 10^−5^
PsSpA	0.27 (0.17, 0.64)	0.07	0.03, 0.17	<1 × 10^−8^
AS	0.32 (0.17, 0.58)	0.37	0.16, 0.85	<1 × 10^−7^
**MMP-3** (^Homogeneity *p* value = 4 × 10^−4^)				
HC	16.67 (9.74, 20.64)			
PsC	13.13 (9.52, 18.37)	1.05	1.00, 1.09	0.06
pPsA	16.54 (11.77, 26.84)	1.06	1.01, 1.10	0.02
PsSpA	19.83 (11.32, 29.60)	1.06	1.01, 1.11	0.02
AS	19.73 (14.90, 28.02)	1.06	1.01, 1.11	0.01
**OPG** (^Homogeneity p value = 5 × 10^−8^)				
HC	0.153 (0.116, 0.173)			
PsC	0.165 (0.130, 0.211)	8.80	0.25, 309.42	0.23
pPsA	0.190 (0.118, 0.285)	25.77	0.72, 913.42	0.07
PsSpA	0.165 (0.121, 0.244)	8.72	0.23, 334.50	0.24
AS	0.165 (0.102, 0.221)	4.78	0.12, 198.22	0.41

*95% CI* 95% confidence interval, *AS* ankylosing spondylitis, *HC* healthy control, *PsC* psoriasis without arthritis, *pPsA* peripheral-only PsA, *PsSpA* psoriatic spondyloarthropathy, *Dkk-1* Dickkopf 1, *MMP-3* matrix metalloproteinase 3, *M-CSF* macrophage colony stimulating factor, *OPG* osteoprotegerin

*n* = 143 AS, 41 HC, 108 pPsA, 194 PsC, 117 PsSpA

^Multinomial logistic regression: homogeneity *p* value tests for no difference in the concentration of each biomarker across all five groups (adjusted for sex, age at blood sampling, and the other biomarkers)

*Multinomial logistic regression comparing HC with each of the 4 disease groups (adjusted for sex, age at blood sampling, and other biomarkers)

Men had significantly lower MMP-3 concentrations than women (adjusted odds ratio, OR_adj_ 0.91; 95% CI 0.83, 0.99; *p* < 0.0001). MMP-3 was associated with hsCRP in patients with PsSpA, pPsA and AS. In patients with AS, the ASDAS was positively associated with Dkk-1 (ß 0.07; *p* = 0.003), M-CSF (ß 0.87; *p* = 0.001), and MMP-3 (ß 0.001; *p* = 0.05), and negatively associated with OPG (ß − 1.66; *p* = 0.03). In patients with PsSpA, the ASDAS was positively associated with M-CSF (ß 0.61; *p* = 0.05) only. ASDAS was included in regression models comparing concentrations in the arthritis groups, to adjust for axial disease activity as a potential confounder, acknowledging that peripheral arthritis is also somewhat captured by the ASDAS. Anti-TNF use did not appear to influence the concentrations of these four biomarkers in this cross-sectional analysis.

### Biomarker concentrations in HC compared with each disease group

On multinomial logistic regression, Dkk-1 concentrations were significantly lower in patients with PsC compared with HC (OR_adj_ 0.72 per ng/mL increase in concentration; 95% CI 0.61, 0.86; *p* = 2 × 10^−4^) (Table 2). M-CSF concentrations were significantly lower in patients with pPsA (OR_adj_ 0.14 per ng/mL increase; 95% CI 0.06, 0.32; *p* < 1 × 10^−5^), PsSpA (OR_adj_ 0.07 per ng/ml increase; 95% CI 0.03, 0.17; *p* < 1 × 10^−8^), and AS (OR_adj_ 0.37 per ng/mL increase; 95% CI 0.16, 0.85; *p* < 1 × 10^−7^) compared with HC. MMP-3 concentrations were significantly higher patients with in pPsA (OR_adj_ 1.06 per ng/mL increase; 95% CI 1.01, 1.10; *p* = 0.02), PsSpA (OR_adj_ 1.06 per ng/mL increase; 95% CI 1.01, 1.11; *p* = 0.02), and AS (OR_adj_ 1.06 per ng/mL increase; 95% CI 1.01, 1.11; *p* = 0.01) compared with HC. OPG concentrations were statistically no different in HC compared with any disease group. The test for homogeneity was used to determine whether biomarkers had significantly different effects when disease groups were modelled separately, whilst controlling for sex, age, and other biomarkers. MMP-3 (homogeneity *p* = 4 × 10^−4^) and OPG (homogeneity *p* = 5 × 10^−8^) concentrations were significantly different across the five groups (Table 2). Taken together, these analyses indicate that Dkk-1, M-CSF, and MMP-3 have different concentrations in healthy controls compared with disease groups, and therefore may be biomarkers of pathology.

### Biomarkers of disease phenotypes

#### Biomarkers of arthritis in patients with psoriasis

Biomarker concentrations were compared in patients with psoriasis with (PsA; *n* = 244) and without (PsC; *n* = 200) inflammatory arthritis. MMP-3 concentrations were significantly higher in patients with PsA (median 17.44 ng/mL; IQR 11.79, 26.88) compared with PsC (median 13.13 ng/mL; IQR 9.52, 18.37) (OR_adj_ 1.02 per ng/mL increase; 95% CI 1.01, 1.03; *p* = 0.0004) (Table 3). Patients with PsA (median 0.28 ng/mL; IQR 0.16, 0.67) had significantly lower M-CSF concentrations than PsC (median 0.62 ng/mL; IQR 0.25, 0.88) (OR_adj_ 0.44 per ng/mL increase in PsA vs. PsC; 95% CI 0.24, 0.82; *p* = 0.01).

**Table 3:** Serum bone biomarker concentrations compared between disease groups

	Unadjusted			Adjusted		
	Odds ratio per ng/mL increase in concentration	95% CI	*p* value	odds ratio per ng/mL increase in concentration	95% CI	*p* value
	**PsA vs. PsC cases**					
Dkk-1	1.16	1.03, 1.32	0.02	1.14*	0.99, 1.31	0.07
M-CSF	0.23	0.13, 0.39	<1 × 10^−7^	0.44*	0.24, 0.82	0.01
MMP-3	1.01	1.00, 1.03	0.03	1.02*	1.01, 1.03	4 × 10^−4^
OPG	2.36	0.80, 6.92	0.12	2.51*	0.68, 9.28	0.17
	**RAD vs. non-RAD cases**					
Dkk-1	1.23	1.06, 1.44	0.01	1.22**	1.05, 1.42	0.01
M-CSF	0.60	0.31, 1.17	0.13	0.64**	0.32, 1.26	0.19
MMP-3	1.01	1.00, 1.02	0.20	1.00**	1.00, 1.01	0.30
OPG	0.17	0.05, 0.62	0.01	0.20**	0.05, 0.80	0.02
	**AS vs. PsSpA cases**					
Dkk-1	1.15	1.01, 1.30	0.04	1.18****	1.02, 1.35	0.02
M-CSF	1.16	0.50, 2.78	0.72	1.28****	0.52, 3.22	0.59
MMP-3	1.00	1.00, 1.01	0.24	1.00****	1.00, 1.01	0.17
OPG	0.22	0.03, 1.54	0.13	0.31****	0.04, 2.27	0.25
	**PsSpA vs. pPsA cases**					
Dkk-1	1.08	0.95, 1.23	0.26	1.06***	0.95, 1.19	0.28
M-CSF	0.55	0.25, 1.21	0.14	0.50***	0.22, 1.11	0.09
MMP-3	1.01	0.99, 1.02	0.31	1.00***	0.99, 1.02	0.73
OPG	0.30	0.08, 1.15	0.08	0.28***	0.06, 1.18	0.08

All models were adjusted potentially for sex, age, disease duration, anti-TNF use, and where appropriate psoriasis, Ankylosing Spondylitis Disease Severity Score (ASDAS), body mass index and RAD)

*PsSpA* psoriatic spondyloarthritis (*n* = 118), *PsA* psoriatic arthritis (*n* = 201), *pPsA* peripheral-only PsA (*n* = 127), *AS* ankylosing spondylitis (*n* = 157), *RAD* radiographic axial disease (*n* = 274), *Dkk-1* Dickkopf 1, *OPG* osteoprotegerin, *MMP-3* matrix metalloproteinase 3, *M-CSF* macrophage colony stimulating factor, *95% CI* 95% confidence interval

*Multivariable logistic regression (reduced model adjusted for age, disease duration, and the Psoriatic Arthritis Spondylitis Radiology Index

**Multivariable logistic regression (reduced model adjusted for sex, age, and disease duration)

***Multivariable logistic regression (reduced model adjusted for the ASDAS)

****Multivariable logistic regression (reduced model adjusted for sex, age, and disease duration)

#### Biomarkers of radiographic axial disease

Biomarker concentrations were compared in patients with arthritis with radiographic axial disease (RAD; *n* = 274; 117 PsSpA and 157 AS) and without RAD (non-RAD; *n* = 127 pPsA). Dkk-1 concentrations were significantly higher in RAD (median 3.42 ng/mL; IQR 2.75, 4.49) compared with non-RAD (median 3.03 ng/mL; IQR 1.93, 3.69) cases (OR_adj_ 1.22 per ng/mL increase; 95% CI 1.05, 1.42; *p* = 0.01) (Table 3). OPG concentrations were significantly lower in patients with RAD (median 0.16 ng/mL; IQR 0.11, 0.22) compared with non-RAD (median 0.19 ng/mL; IQR 0.12, 0.28) (OR_adj_ 0.20 per ng/mL increase; 95% CI 0.05, 0.80; *p* = 0.02). Skin psoriasis was not a significant covariable.

Biomarkers were tested along a spectrum of increasing RAD. Patients with AS had significantly higher Dkk-1 concentrations than those with PsSpA (OR_adj_ 1.18 per ng/mL increase; 95% CI 1.02, 1.35; *p* = 0.02) (Table 3). Biomarker concentrations were statistically no different in patients with PsSpA and patients with pPsA.

### Association of phenotype with a biomarker panel

Multivariable logistic regression was performed to determine whether a combination (panel) of bone biomarkers and hsCRP, excluding clinical covariables, might have a stronger association with disease phenotype. A number of different panels were tested. The panel comprising Dkk-1 (OR_adj_ 0.09 per ng/mL increase; 95% CI 0.03, 0.23; *p* = 4 × 10^−7^), MMP-3 (OR_adj_ 1.07 per ng/mL increase; 95% CI 1.02, 1.12; *p* = 0.002) and OPG (OR_adj_ 31.00 per ng/mL increase; 95% CI 0.66, 1451.86; *p* = 0.08) was best able to differentiate patients with PsA from HC, with an area under the receiver operating characteristic (ROC) curve (AUC) of 0.84. The AUC was < 0.73 for other all other biomarker panels as determinants of phenotype (PsA vs. PsC, PsC vs. HC, RAD vs. non-RAD, PsSpA vs. pPsA, and PsSpA vs. AS).

### Biomarkers of radiographic axial disease severity and morphology

Using multivariable generalised additive models, no biomarker was associated with RAD severity (as measured by the mSASSS and PASRI) or morphology (as measured by the osteoproliferation subdomain of the PASRI, which scores for vertebral corner sclerosis, vertebral syndesmophyte formation, and cervical facet joint fusion) in either PsSpA or AS. In patients with PsSpA only, Dkk-1 concentrations were significantly lower in patients with a PASRI erosion score ≥ 1 (OR_adj_ 0.28 per ng/mL increase; 95% CI 0.10, 0.80; *p* = 0.02) than in those without erosions*.*

### Biomarker concentration thresholds differentiating disease groups

ROC analyses were performed to determine whether MMP-3 and M-CSF concentration thresholds could usefully differentiate patients with PsA from HC. In a model of maximum accuracy (proportion of cases correctly classified), an MMP-3 threshold of 7.21 ng/mL had sensitivity of 0.99 and specificity of 0.12 (AUC 0.66; 95% CI 0.59, 0.74), and an M-CSF threshold of 0.02 ng/mL had sensitivity of 1.00 and specificity of 0.00 (AUC 0.24; 95% CI 0.17, 0.31) (Table 4).

**Table 4:** Biomarker concentration thresholds differentiating disease groups, using receiver operating characteristic analyses

	Maximum accuracy(proportion of cases correctly classified)		Minimum value forsensitivity ≥ 0.95	
	**PsC vs. PsA**	**HC vs. PsA**	**PsC vs. PsA**	**HC vs. PsA**
	*MMP-3*			
Threshold (ng/mL)	14.51	7.21	8.22	8.32
Sensitivity	0.76	0.99	0.96	0.95
Specificity	0.60	0.12	0.16	0.20
AUC (95% CI)	0.70 (0.65, 0.75)	0.66 (0.59, 0.74)	0.70 (0.65, 0.75)	0.66 (0.59, 0.74)
	*M-CSF*			
Threshold (ng/mL)	0.05	0.02	0.10	0.02
Sensitivity	0.99	1.00	0.95	1.00
Specificity	0.01	0.00	0.02	0.00
AUC (95% CI)	0.34 (0.29, 0.40)	0.24 (0.17, 0.31)	0.34 (0.29, 0.40)	0.24 (0.17, 0.31)
	**PsA cases: Non-RAD vs. RAD**		**PsA cases: Non-RAD vs. RAD**	
	*Dkk-1*			
Threshold (ng/mL)	4.96		0.91	
Sensitivity	0.15		0.96	
Specificity	0.94		0.05	
AUC (95% CI)	0.56 (0.44, 0.67)		0.56 (0.44, 0.67)	
	*OPG*			
Threshold (ng/mL)	3.88		0.08	
Sensitivity	0.00		0.95	
Specificity	0.99		0.08	
AUC (95% CI)	0.47 (0.39, 0.56)		0.47 (0.39, 0.56)	

*AUC* area under the curve, *95CI* 95% confidence interval, *Dkk-1* Dickkopf 1, *M-CSF* Macrophage colony stimulating factor, *MMP-3* Matrix metalloproteinase 3, *OPG* osteoprotegerin, *PsA* psoriatic arthritis, *PsC* psoriasis without arthritis, *HC* healthy control, *RAD* radiographic axial disease

Similarly, ROC analyses were used to determine whether MMP-3 and M-CSF concentration thresholds could usefully differentiate PsA from PsC. In a model of maximum accuracy an MMP-3 threshold of 14.51 ng/mL had sensitivity of 0.76 and specificity of 0.60 (AUC 0.70; 95% CI 0.65, 0.75); and an M-CSF threshold of 0.05 ng/ml had sensitivity of 0.99 and specificity of 0.01 (AUC 0.34; 95% CI 0.29, 0.40). The effect of mandating sensitivity of > 0.95, possibly at the expense of specificity, is shown in Table 4.

Since in patients with PsA (*n* = 200), Dkk-1 concentrations were higher and OPG concentrations were lower in patients with with RAD compared to those without RAD, biomarker concentration thresholds could potentially be used to identify patients with PsA for spinal imaging. In a model of maximum accuracy, a Dkk-1 threshold of 4.96 ng/mL had sensitivity of 0.15 and specificity of 0.94 (AUC 0.56; 95% CI 0.44, 0.67); and an OPG threshold of 3.88 ng/ml had sensitivity of 0.00 and specificity of 0.99 (AUC 0.47; 95% CI 0.39, 0.56) (Table 4). The effect of mandating sensitivity of > 0.95, possibly at the expense of specificity, is shown in Table 4.

## Discussion

We have previously reported a study of the same cohort, showing clinical, imaging, and genetic signatures unique to pPsA, PsSpA, and AS [2]. Using the same large cohort of well-characterised cases and matched healthy controls, we sought to determine whether there are unique serum-soluble bone-turnover biomarker signatures. Previously reported studies that have investigated PsA in terms of serum bone biomarkers have been limited by small sample size, variable endpoints, and heterogenous laboratory methods [1].

MMP-3 and M-CSF appear to be biomarkers of arthritis, differentiating patients with PsA from those with PsC, and patients with SpA from HC. Hence, MMP-3 and M-CSF concentration thresholds may be useful to screen for PsA in patients with PsC. We have presented two such models, allowing the clinician to choose whether very high sensitivity at the expense of specificity is most important in their setting (model with ≥ 0.95 sensitivity), or a more equitable balance between sensitivity and specificity is required (model of maximum accuracy). Neither MMP-3 nor M-CSF differentiated various forms of SpA, i.e. pPsA, PsSpA and AS, implying they may share a common pathological pathway, e.g. entheseal disease or bone resorption. Other studies have shown MMP-3 levels to be four times higher in synovial fluid compared with serum in patients with AS with peripheral involvement [11], and a thousand times higher in synovial tissue compared with serum in patients with SpA with peripheral involvement [12]. MMP-3 levels are higher in patients with axial SpA with peripheral arthritis rather than without [12–14]. MMP-3 may therefore be a biomarker more specific to peripheral synovial-based arthritis, than to axial non-synovial entheseal-based arthritis. MMP-3 and M-CSF need further testing to determine their performance in differentiating PsA from rheumatoid arthritis (RA) and inflammatory osteoarthritis of the interphalangeal joints.

Dkk-1 appears to be a biomarker of axial disease in SpA, with a pattern for increasing concentration along a spectrum of increasing axial arthritis. Dkk-1 could be used to differentiate patients with PsA with and without axial arthritis, and patients with PsSpA from those with AS. Our previous research has shown that 25% of patients with PsA with radiographic axial disease do not recall ever having inflammatory axial symptoms [2]. Dkk-1 and OPG testing may therefore offer an opportunity to identify “symptomatically-silent” axial disease in PsA. We have therefore proposed Dkk-1 concentration thresholds that might be used to screen for axial disease in patients with PsA. Similarly, OPG appears to be a biomarker of axial disease in patients with SpA, and could be used to differentiate patients with axial SpA from peripheral-only SpA, independently of psoriasis status.

Since Dkk-1 is an inhibitor of the Wnt pathway, which normally induces osteoblastogenesis and new bone formation, one might expect Dkk-1 concentrations to be progressively lower along a spectrum of diseases with increasing new bone formation. However, consistent with most other studies, Dkk-1 concentrations were higher in patients with AS compared with HC [15–17]; no different in patients with pPsA compared with HC [15]; and higher in patients with AS compared with PsA [15]. Dkk-1 levels may be higher in SpA, particularly AS, because Dkk-1 is pathologically dysfunctional. Daoussis et al. found that whilst serum total Dkk-1 levels are higher in patients with AS compared with HC or patients with PsA, Dkk-1 is dysfunctional in AS; Dkk-1 binds less avidly to its receptor LRP6, has an abnormal stimulatory effect on the Wnt pathway, and responds abnormally to anti-Dkk-1 monoclonal antibodies [15]. Yucong et al. also reported less avid binding of Dkk-1 to its receptor in patients with AS compared with HC [18]. These studies explain the progressively higher Dkk-1 concentrations along our spectrum of patients with increasing RAD, as an attempt to compensate for dysfunctional Dkk-1. Dysfunctional Dkk-1 is inadequately inhibiting Wnt-pathways, allowing unimpeded bone formation, possibly translating to the osteoproliferative phenotype of SpA.

We found lower Dkk-1 concentrations in patients with PsSpA with vertebral erosions. In human embryonic stems cells, Dkk-1 plays an important role promoting synovial angiogenesis, that might encourage inflammatory pannus formation, and subsequent erosion [19]. There is emerging evidence that Wnt-pathways are involved in non-bone pathways associated with psoriasis, characterised by keratinocyte hyperproliferation and altered innate immunity [20–25].

In SpA there is a paradox of osteoproliferation and bone resorption. Patients with AS [26–28] or PsA [29] are prone to osteoporosis. As OPG inhibits bone resorption, low OPG levels may translate to low bone mineral density. However, as we did not measure bone mineral density, we cannot determine if these serum biomarkers are reflecting SpA-related pathology in trabecular bone, or vertebral corners and sacroiliac joints. The measurement of these bone markers in tissues sampled from the vertebral corners and sacroiliac joints would be mechanistically more informative. However, obtaining such samples in live participants would be procedurally challenging, painful for participants, and likely hamper study enrolment. Our study would have been further strengthened had we measured serum RANKL levels, as there is some evidence that the ratio of RANKL:OPG is more indicative of axis dysregulation than either biomarker alone [30–33].

Further research is needed to determine whether synthetic and biological DMARDs directly alter bone biomarkers, independently of their influence on disease activity. Within the constraints of our cross-sectional study design, we demonstrated no relationship between anti-TNF use and levels of biomarkers. However, other studies have shown that biomarker concentrations are influenced by anti-TNF use, longitudinally in PsA [34, 35], cross-sectionally [32, 36] and longitudinally [11, 17, 37–40] in AS, in AS clinical responders and non-responders [38], only in AS patients with peripheral arthritis [41], and with differential direction of change (Dkk-1) in AS compared with RA [15].

The reliability of our results is strengthened by the large sample size, robust case classification, no missing data for three biomarkers, matching of HCs with PsA cases, and multivariable regression modelling allowing adjustment for confounders, particularly disease duration and activity. Our results are generalisable to real-world clinical practice, because consecutive unselected clinic attendees were invited to participate, reducing selection bias, and enrolling patients of differing ages, stages of disease, and disease activity.

Our results would have been strengthened had reliable commercially available kits been available to measure active-MMP-3 rather than total MMP-3 [42–45], functional Dkk-1 or Dkk-1 biological activity rather than total Dkk-1 [15, 18, 46], RANK ligand [30–33], sclerostin, and neoepitopes of type 2 collagen metabolism (CPII and C2C). The cross-sectional design of our study reduced the ability to entirely adjust for time-varying variables such as disease activity, medication use and BMI, and unmeasured confounders known to alter OPG and MMP-3 concentrations [10, 47, 48]. However, the direction and magnitude of their confounding is unlikely to be significantly different across the four disease groups. We acknowledge that some patients with pPsA will have non-radiographic PsSpA, perhaps better detected on magnetic resonance imaging (MRI) or computed tomography (CT).

## Conclusions

This study gives further insight into the pathophysiology of psoriasis, PsA, and AS. These bone biomarkers offer potential to screen for PsA in psoriasis cohorts, screen for axial arthritis in PsA, and help differentiate PsSpA from AS. A larger study is underway by our group to replicate these findings in an independent well-characterised inception cohort. These four markers will also be investigated longitudinally as biomarkers of disease activity, treatment response, and prognosis.

## Abbreviations

ADIPSA: Axial Disease in Psoriatic Arthritis study
AS: Ankylosing spondylitis
ASDAS: Ankylosing Spondylitis Disease Activity Score
AUC: Area under the curve
BMI: Body mass index
CASPAR: Classification of Psoriatic Arthritis
Dkk-1: Dickkopf-1
ELISA: enzyme-linked immunosorbent assay
HC: Healthy controls
hsCRP: High-sensitivity C-reactive protein
M-CSF: Macrophage-colony stimulating factor
MMP-3: Matrix metalloproteinase-3
mSASSS: Modified Stoke Ankylosing Spondylitis Spinal Score
OPG: Osteoprotegerin
PASRI: Psoriatic Arthritis Spondylitis Radiology Index
pPsA: Psoriatic arthritis without axial arthritis
PsA: Psoriatic arthritis
PsC: Psoriasis without arthritis
PsSpA: Psoriatic spondyloarthritis
RA: Rheumatoid arthritis
RAD: Radiographic axial disease
ROC: Receiver operating characteristic
SIJ: sacroiliac joint
TNF: Tumour necrosis factor
